# Base-Resolution Analysis of DNA Methylation Patterns Downstream of *Dnmt3a* in Mouse Naïve B Cells

**DOI:** 10.1534/g3.117.300446

**Published:** 2018-01-11

**Authors:** Christopher G. Duncan, Hrisavgi D. Kondilis-Mangum, Sara A. Grimm, Pierre R. Bushel, Kaliopi Chrysovergis, John D. Roberts, Frederick L. Tyson, B. Alex Merrick, Paul A. Wade

**Affiliations:** * Epigenetics and Stem Cell Biology Laboratory, National Institute of Environmental Health Sciences, Research Triangle Park, North Carolina 27709; † Integrative Bioinformatics, National Institute of Environmental Health Sciences, Research Triangle Park, North Carolina 27709; ‡ Biostatistics and Computational Biology Branch, National Institute of Environmental Health Sciences, Research Triangle Park, North Carolina 27709; § Division of Extramural Research and Training, National Institute of Environmental Health Sciences, Research Triangle Park, North Carolina 27709; ** Division of the National Toxicology Program, National Institute of Environmental Health Sciences, Research Triangle Park, North Carolina 27709

**Keywords:** B cell, DNA methylation, *Dnmt3a*, whole genome bisulfite sequencing, gene body

## Abstract

The DNA methyltransferase, *Dnmt3a*, is dynamically regulated throughout mammalian B cell development and upon activation by antigenic stimulation. *Dnmt3a* inactivation in hematopoietic stem cells has been shown to drive B cell-related malignancies, including chronic lymphocytic leukemia, and associates with specific DNA methylation patterns in transformed cells. However, while it is clear that inactivation of *Dnmt3a* in hematopoietic stem cells has profound functional effects, the consequences of *Dnmt3a* inactivation in cells of the B lineage are unclear. To assess whether loss of *Dnmt3a* at the earliest stages of B cell development lead to DNA methylation defects that might impair function, we selectively inactivated *Dnmt3a* early in mouse B cell development and then utilized whole genome bisulfite sequencing to generate base-resolution profiles of *Dnmt3a^+/+^* and *Dnmt3a^−/−^* naïve splenic B cells. Overall, we find that global methylation patterns are largely consistent between *Dnmt3a^+/+^* and *Dnmt3a^−/−^* naïve B cells, indicating a minimal functional effect of DNMT3A in mature B cells. However, loss of *Dnmt3a* induced 449 focal DNA methylation changes, dominated by loss-of-methylation events. Regions found to be hypomethylated in *Dnmt3a^−/−^* naïve splenic B cells were enriched in gene bodies of transcripts expressed in B cells, a fraction of which are implicated in B cell-related disease. Overall, the results from this study suggest that factors other than *Dnmt3a* are the major drivers for methylome maintenance in B cell development.

DNA methylation is an essential epigenetic modification in mammals (Li *et al.* 1992; Okano *et al.* 1999). Cytosine methylation, occurring predominantly in the context of CpG dinucleotides in mammalian cells, has long been hypothesized to play a critical role in the establishment and maintenance of cell type-specific gene expression (Holliday and Pugh 1975; Riggs 1975). Indeed, epigenetic modifications, including DNA methylation, are dynamically regulated throughout hematopoietic differentiation (Cabezas-Wallscheid *et al.* 2014; Lara-Astiaso *et al.* 2014). Moreover, massive perturbation of the DNA methylome occurs during B cell differentiation, maturation, and activation (Kulis *et al.* 2015; Lai *et al.* 2013; Barwick *et al.* 2016; Oakes *et al.* 2016).

The DNA methyltransferases (DNMTs), DNMT1, DNMT3A, DNMT3B, and DNMT3C establish and maintain DNA methylation patterns in mammalian cells (Jones and Liang 2009; Barau *et al.* 2016). Accordingly, DNMTs are dynamically regulated in B cell maturation and activation. We have previously demonstrated significant changes in expression of *DNMT1*, *DNMT3A*, and *DNMT3B*, concomitant with widespread alteration of the DNA methylation landscape, throughout the course of a T cell-dependent B cell immune response (Lai *et al.* 2013). Upon activation by antigen, *DNMT1* and *DNMT3B* were upregulated (Lai *et al.* 2013), consistent with the role of DNMT1 in replication-dependent maintenance of DNA methylation (Leonhardt *et al.* 1992) and with the role of DNMT3B in late-stage B cell differentiation (Blanco-Betancourt *et al.* 2004). In contrast, *DNMT3A* is dramatically decreased upon activation by antigen (Lai *et al.* 2013). Correspondingly, extensive DNA methylation changes were observed upon activation by antigenic stimulation, between naïve and germinal center (GC) B cell populations, and the alterations were dominated by loss-of-methylation events. These site-specific DNA methylation changes were hypothesized to result from passive (rather than active) demethylation associated with a coordinated loss of DNMT3A levels, a massive burst of proliferation, and widespread alteration of nuclear architecture (Lai *et al.* 2013). However, the precise role of *Dnmt3a* in directing DNA methylation patterns in naïve B cells has not been characterized.

In addition to roles in normal B cell development, experimental and human sequencing data point to a role for *DNMT3A* mutation and/or loss-of-function in hematologic disease. *DNMT3A* is one of the most commonly mutated genes in adult hematologic malignancies (Brunetti *et al.* 2017; Yang *et al.* 2015). Loss of *Dnmt3a* progressively impairs hematopoietic stem cell differentiation (Challen *et al.* 2011) and confers a preleukemic phenotype on murine hematopoietic stem cells (Mayle *et al.* 2015). Further, inactivation of *Dnmt3a* in mouse hematopoietic stem cells induces chronic lymphocytic leukemia (CLL) and CD8-positive peripheral T cell lymphomas (Haney *et al.* 2016a,b; Peters *et al.* 2014). In transformed cells, *Dnmt3a* mutations and loss-of-function associate with specific DNA methylation patterns. For instance, *DNMT3A* mutations are associated with a specific DNA hypomethylation pattern in acute myeloid leukemia (Russler-Germain *et al.* 2014), and loss of DNMT3A leads to hypomethylation of hematopoietic enhancers in FLT3-ITD–associated leukemias (Yang *et al.* 2016). Accordingly, a cell type-specific function has been suggested for DNMT3A in cellular transformation (Haney *et al.* 2016a). However, while it is clear that inactivation of *Dnmt3a* at the hematopoietic stem cell stage has profound functional effects, the consequences of *Dnmt3a* inactivation in cells of the B lineage are unclear.

Here, we assess whether loss of *Dnmt3a* at the earliest stages of B cell development lead to DNA methylation defects that might impair function. We selectively inactivated *Dnmt3a* early in B cell development and then utilized whole genome bisulfite sequencing (WGBS) to characterize global DNA methylation patterns downstream of *Dnmt3a* in splenic naïve B cells. Overall, we find that global methylation patterns are largely consistent between *Dnmt3a^+/+^* and *Dnmt3a^−/−^* naïve B cells, indicating a minimal functional effect of DNMT3A in mature B cells. However, loss of *Dnmt3a* induced a small number of focal DNA methylation changes, and the differentially methylated regions (DMRs) were found to be enriched in and around genes that are expressed in B cells and that are implicated in B cell-related disease.

## Materials and Methods

### Mouse lines and breeding strategy

*Dnmt3a^flox/flox^* mice containing conditional alleles for *Dnmt3a* (B6;129-*Dnmt3a^tm1Jae^*/Mmnc, RRID:MMRRC_031042-UNC) (Nguyen *et al.* 2007) were obtained from the Mutant Mouse Resource Research Centers. *Cd19^Cre^* mice containing the CD19-Cre allele (B6.129P2(C)-*Cd19^tm1(cre)Cgn^*/J, RRID:IMSR_JAX:006785) (Rickert *et al.* 1997) were obtained from The Jackson Laboratory. *Dnmt3a^flox/flox^* animals were bred with *Cd19^Cre^* animals to generate *Dnmt3a^flox/+^* × *Cd19^Cre/+^* progeny. F1 *Dnmt3a^flox/+^* × *Cd19^Cre/+^* animals were further bred to maintain *Dnmt3a^flox/flox^* × *Cd19^Cre/+^* (*Dnmt3a^−/−^*) and *Dnmt3a^flox/flox^* × *Cd19^+/+^* (*Dnmt3a^+/+^*) littermate control mice. Animals were housed in a specific pathogen-free environment at the National Institute of Environmental Health Sciences (NIEHS). All animal experiments were approved by the NIEHS Institutional Animal Care and Use Committee and were performed according to National Institutes of Health guidelines for the care and use of laboratory animals. All procedures were in compliance with the Animal Welfare Act Regulations 9 CFR 1-4, with handling and treatment according to the *Guide for the Care and Use of Laboratory Animals* [National Research Council (US) 2011].

### Splenic naïve B cell isolation

Spleen was harvested from three male *Dnmt3a^−/−^* and three male *Dnmt3a^+/+^* mice, ranging from 2 to 4 months of age, following euthanasia by carbon dioxide asphyxiation. Single cell suspensions were prepared and cells were blocked with a cocktail of rat IgG (Jackson ImmunoResearch Laboratories) and anti-mouse CD16/CD32 (2.4G2) on ice for 10 min. Cells were then stained with allophycocyanin-B220 (clone RA3-6B2), phycoerythrin (PE)-IgD (clone 11-26c.2a), and FITC-GL7 (clone GL7) from BioLegend, eBioscience, or BD Pharmingen for 30 min in the dark. Cells were subsequently washed two times and then sorted on a BD FACSAria cell sorter (BD Biosciences). B220^+^IgD^++^GL7^−^ B cells were sorted to a purity of 98–99%. Deletion efficiency of *Dnmt3a* was assessed by quantitative PCR, performed using SYBR Green on a CFX Connect Real-Time PCR Detection System (BioRad).

### Quantification of lymphocyte populations in bone marrow and spleen

Bone marrow and spleen were harvested from three male *Dnmt3a^−/−^* and three male *Dnmt3a^+/+^* mice following euthanasia by carbon dioxide asphyxiation. Single cell suspensions were prepared and cells were blocked with a cocktail of rat IgG (Jackson ImmunoResearch Laboratories) and anti-mouse CD16/CD32 (2.4G2) on ice for 10 min. Cells from bone marrow were stained with antibodies against mouse B220 (RA3-6B2), CD43 (S7), and IgM; splenocytes were stained with antibodies against mouse B220, CD19 (1D3), CD4 (GK1.5), and CD8a (53-6.7). All antibodies were purchased from BioLegend, eBioscience, Southern BioTech, or BD Pharmigen. Cells were subsequently washed two times and then analyzed on a BD LSR II Flow Cytometer (BD Biosciences) or sorted on FACSAria cell sorter (BD Biosciences). The following cell populations were analyzed from bone marrow: pro B cells (B220^+^CD43^+^IgM^−^), pre B cells (B220^+^CD43^−^IgM^−^), and immature B cells (B220^+^CD43^−^IgM^+^). The following cell populations were analyzed from spleen: CD4^+^ T cells (B220^−^CD19^−^CD4^+^CD8^−^), CD8^+^ T cells (B220^−^CD19^−^CD4^−^CD8^+^), B cells (B220^+^CD19^+^CD4^−^CD8^−^), and nonlymphocytes (B220^−^CD19^−^CD4^−^CD8^−^). Cell populations were sorted to a purity of 98–99%.

### Nucleic acid extraction and purification

Sorted B220^+^IgD^++^GL7^−^ B cells were homogenized in TRIzol (Thermo Fisher Scientific), and phase separation was performed according to the manufacturer’s instructions. Genomic DNA was extracted from the TRIzol organic phase by adding an equal volume of back extraction buffer, consisting of 4 M guanidine thiocyanate, 50 mM sodium citrate, and 1 M Tris base, and precipitated with isopropanol. DNA was further purified by performing phenol:chloroform:isoamyl alcohol (25:24:1) extractions (Ambion) according to the manufacturer’s instructions. DNA integrity was assessed by gel electrophoresis.

### WGBS library construction and sequencing

Quantification of DNA during library production was performed using Qubit dsDNA Assay Kits (Invitrogen). For each sample, 1500 ng intact genomic DNA and 1.5 ng unmethylated λ DNA (Promega) were added to a microTUBE (Covaris) and fragmented using a Covaris S220 focused ultrasonicator according to the manufacturer’s instructions, with the following settings: target BP (peak), 300; peak incident power (W), 175; duty factor, 10%; cycles per burst, 200; treatment time (sec), 50; temperature (°C), 7; water level, 12; and sample volume (microliter), 50. Sequencing libraries were prepared using the NEXTflex Bisulfite-Seq Kit (BIOO Scientific) with a starting input of 1 μg. Steps for end repair, adenylation, and adapter ligation were performed according to the manufacturer’s instructions, following “option 1” and using NEXTflex Bisulfite-Seq Barcodes. Agarose gel size selection was implemented by electrophoresing the sample on a 2% NuSieve 3:1 agarose gel (Lonza) at 120 V for 10 min and then at 60 V for 120 min. The ladder lane was excised and visualized on a UV transilluminator, and then a gel slice was cut from each sample corresponding to 410–510 bp using a clean razor. DNA was recovered using a QIAquick Gel Extraction Kit (Qiagen) and then cleaned using an equal volume of AMPure XP (Agencourt). Bisulfite conversion was performed using an EpiTect Bisulfite Kit (Qiagen) according to the manufacturer’s instructions with the following modified thermal cycler conditions: 5 min at 95°, 25 min at 60°, 5 min at 95°, 85 min at 60°, 5 min at 95°, 175 min at 60°, 5 min at 95°, 180 min at 60°, 5 min at 95°, and 180 min at 60°, then hold at 20°. After elution, the samples were cleaned using an equal volume of AMPure XP (Agencourt). Bisulfite converted DNA product was PCR amplified for 10 cycles using KAPA HiFi Uracil^+^ Ready Mix (Kapa Biosystems) according to the manufacturer’s protocol. Following amplification, the libraries were cleaned using an equal volume of AMPure XP (Agencourt). The size of each amplified library was validated on Bioanalyzer High Sensitivity DNA Chips (Agilent Technologies). After library construction, all six libraries were equally multiplexed and sequenced using the Illumina NextSeq500 platform in paired-end format for 76 or 151 cycles at the NIEHS Epigenomics Core Laboratory.

### Read processing and alignment

Raw FASTQ datasets were trimmed to remove the final low quality base (to 150 or 75 bp) and filtered to include read pairs with average base quality score of at least 20. Trimmed and filtered read pairs were aligned to the mouse (mm9/NCBI build 37) and phage λ (NC_001416.1) genomes using Bismark version 0.14.0 (Krueger and Andrews 2011) with bowtie version 0.12.8 (Langmead *et al.* 2009), with parameters “-X 10000--unmapped--non_bs_mm--old_flag -n 2 -l 50 -e 70--chunkmbs 1024.” A second-pass mapping step was implemented such that unmapped reads from the PE150 mapping were trimmed to a read length of 75 bp, and trimmed reads were re-submitted to Bismark as described above. For each mapped SAM file, positional bias (M-bias) was assessed using “bismark_methylation_extractor” with the following options: “--paired-end--mbias_only--include_overlap.” Positional bias trim positions were defined by first calculating the mid-read mean and SD of positional CpG methylation level for each individual read using positions 30–100 for PE150 reads and positions 20–50 for PE75 reads. Positions were retained according to the following criteria: (1) retain positions with CpG methylation level within ±3 SD of mid-read mean, (2) range must start and end with positions that meet the ±3 SD of mid-read mean criteria, and (3) single (nonconsecutive) outliers allowed as long as within ±4 SD of mid-read mean. All SAM files per biological replicate were merged then deduplicated using the Bismark “deduplicate_bismark” tool with the “--paired” option. Following deduplication, positional bias trimming was implemented, and overlapping mates were clipped. Mapped reads were split by genome (mm9/λ) and strand (plus/minus). For each library, bisulfite conversion rate was assessed using reads mapping to (1) unmethylated phage λ spike-in and (2) mitochondrial DNA (chrM). Composite bisulfite conversion rates were at least 99.84% for all libraries, and results were highly consistent between phage λ and chrM reads. Postprocessed BAM files for biological replicates (three replicates per group) were then merged using “MergeSamFiles” of Picard Tools, keeping strands separate. Sequence alignment and processing metrics are summarized in Supplemental Material, Table S1 of File S1.

### Genomic context validation for CpG sites

For downstream analysis of CpG methylation, a genomic context validation strategy was implemented to identify mm9 CpG sites that are within a CpG context in all samples. Strand-specific data from all runs of all samples were combined, and each CpG dinucleotide in mm9 was assessed using reads mapped to the G on the strand opposite each cytosine. For a CpG site to be retained for downstream methylation analyses, the following criteria must be met, where N represents the cytosine position of the plus strand: (1) at least 95% of mapped bases at the N + 1 coordinate on the plus stand (opposite the expected cytosine on the minus strand) must be G, (2) at least 95% of mapped bases at the N coordinate on the minus strand (opposite the expected cytosine on the plus strand) must be G, (3) the minimum G read depth at the N + 1 position on the plus strand must be at least 5, and (4) the minimum G read depth at the N position on the minus strand must be at least 5. A total of 18,312,876 out of 21,722,957 (84.3%) CpG sites passed the genomic context validation criteria and were utilized for downstream differential methylation analyses.

### Comparative methylation analyses

For comparative analysis of single-site CpG methylation levels a read depth filter was applied such that only validated CpGs with at least 10 reads were included. Differential methylation analyses were performed using MOABS v1.3.2 (Sun *et al.* 2014). CpG mpileup files for *Dnmt3a^+/+^* and *Dnmt3a^−/−^* were formatted in “*.G.bed” format for input to MOABS. DMRs were identified using the “mcomp” module using default parameters except “–minNominalDif = 0.2.” Pairwise comparisons were reported using method M1. Details regarding genomic coordinates, methylation levels, and statistical significance of DMRs are listed in Tables S2 and S3 of File S1. The smoothed density scatterplot of mCG/CG was generated using the “smoothScatter” function of the “graphics” package in R, which produces a smoothed color density representation of the scatterplot, obtained through a kernel density estimate. Density color keys were generated using the “image.plot” and “tim.colors” functions of the “fields” package. Box and whisker plots of methylation levels were created using the “boxplot” function of the “stats” package using the default settings. Boxes represent the first to third quartiles of the data distribution and whiskers were drawn to the maximum data value no more than 1.5 times the interquartile distance.

### Genomic feature annotation and statistical analyses

Statistical and graphical analyses were conducted using R version 3.3.2 (R Core Team 2016). Genomic feature annotation was performed using the Goldmine R package version 1.0 (Bhasin and Ting 2016). Gene model features were obtained from RefSeq annotations (February 17, 2017) (O’Leary *et al.* 2016). Promoters were defined as the region from 1000 bp upstream to 500 bp downstream of TSSs. Gene 3′ ends were defined as the region from 1000 bp upstream to 1000 bp downstream of TESs. CpG island coordinates for mm9 were obtained from the UCSC Table Browser (Karolchik *et al.* 2004). CpG island shores were defined as regions up to 2 kb flanking either CpG island edge (Doi *et al.* 2009; Irizarry *et al.* 2009). CpG island shelves were defined as regions up to 2 kb flanking CpG island shores (Bibikova *et al.* 2011). Genomic distance of DMRs was calculated to the nearest single TSS using the “closest” utility of Bedtools version 2.23.0 (Quinlan and Hall 2010), reporting each distance with respect to the TSS’s strand. Length-matched random control coordinates were generated using the “shuffle” utility of Bedtools using DMR coordinates with the options “-seed 1000” and “–noOverlapping” and masking mm9 gap regions, noncanonical chromosomes, and chrM. Association of genomic regions with putative target genes and the Mouse Genome Informatics (MGI) Phenotype Ontology (Blake *et al.* 2009) and Disease Ontology (Osborne *et al.* 2009) was carried out using the Genomic Regions Enrichment of Annotations Tool (GREAT version 3.0.0) (McLean *et al.* 2010) with the following parameters: background, whole genome background; assembly, mouse: NCBI build 37 (mm9); associated genomics regions: basal + extension, curated regulatory domains included. Depicted values for statistical enrichments for associations between genomic regions and annotations represent raw *P*-values using a binomial test over genomic regions with a minimum twofold region-based enrichment. For analysis of DMR overlap with bodies of genes expressed in B cells, we used a published RNA-seq dataset from Shi *et al.* (2015), and data were obtained from the NCBI Gene Expression Omnibus (GEO) (Edgar *et al.* 2002) (accession no.: GSE60927). Normalized expression values were obtained for two biological replicates (samples GSM1493786 and GSM1493787) of mouse follicular B cells (small, B220^+^CD21^+^CD23^+^). Genes with an average of at least one fragment per kilobase of transcript per million mapped reads were defined as expressed. EntrezID (gene ID) numbers for expressed genes were converted to RefSeq mRNA accession using the biological DataBase network (bioDBnet) database to database conversion tool (db2db) for *Mus musculus* (taxon ID 10090) (Mudunuri *et al.* 2009). Gene body coordinates for expressed genes, defined as TSS to TES, were obtained from RefSeq annotations (O’Leary *et al.* 2016). For comparison to *Dnmt3a*-mutant CLL WGBS data, gene body and promoter DMRs hypomethylated in Dnmt3a^Δ/Δ^ CLL relative to B-1a were obtained from Haney *et al.* (2016a), and coordinates were converted to mm9 using liftOver. A Z-test for the difference between two proportions was utilized to test whether the proportion of hypo- or hyper-methylated DMRs overlapping a given genomic feature differs from that of a length-matched random null coordinate set.

### Data availability

The datasets generated during the current study are available in the NCBI GEO (Edgar *et al.* 2002) and are accessible through GEO series accession number GSE100262 (https://www.ncbi.nlm.nih.gov/geo/query/acc.cgi?acc=GSE100262). File S1 contains a summary of WGBS processing metrics (Table S1 in File S1) and summaries of hypomethylated DMRs (Table S2 in File S1) and hyper-methylated DMRs (Table S3 in File S1).

## Results

### Targeted knockout of Dnmt3a in mouse B cells

**Figure 1:**
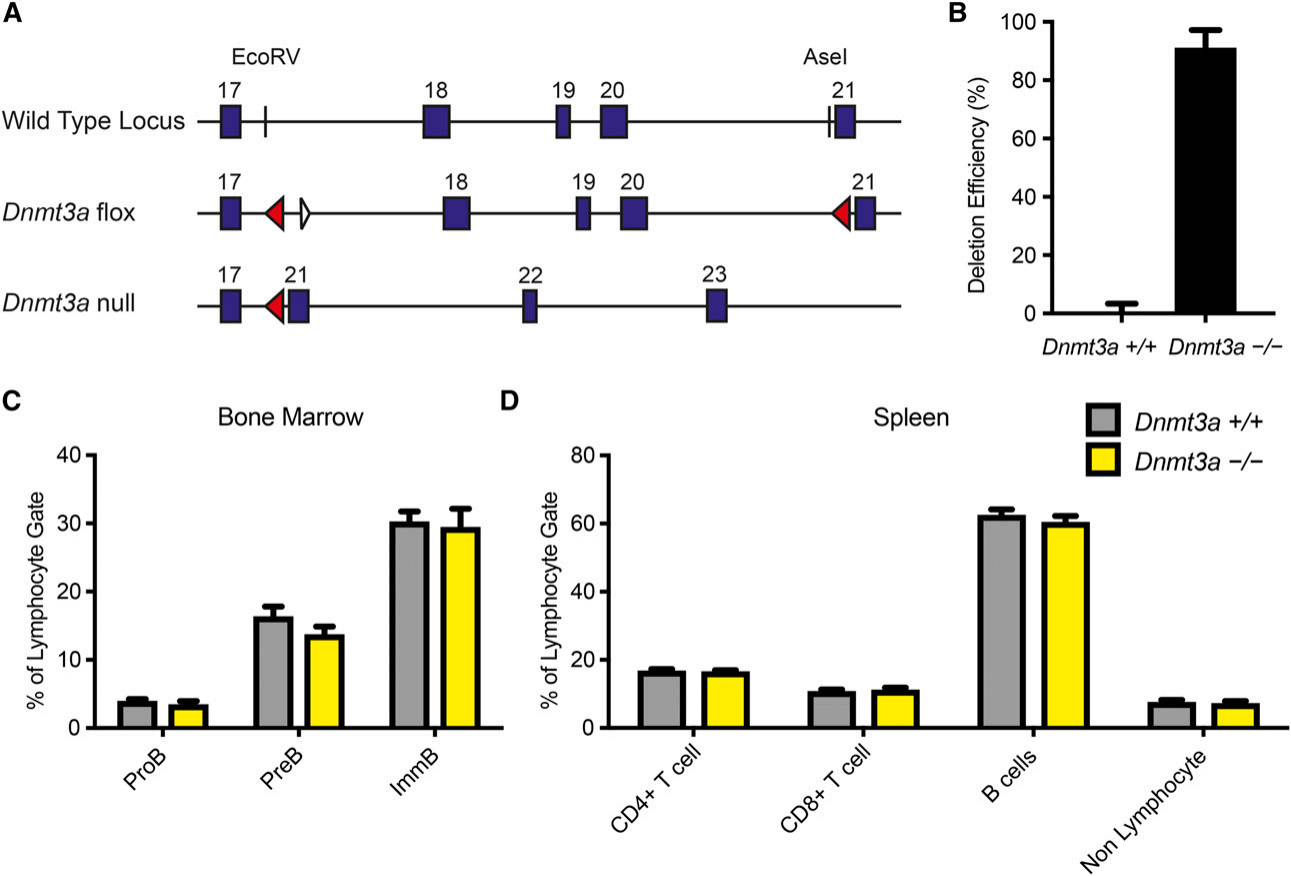
Conditional knockout of *Dnmt3a* in mouse B cells. (A) *Dnmt3a* locus and gene targeting strategies. Top panel: endogenous *Dnmt3a* locus including relative positions of exons (blue boxes) and restriction enzyme sites (*Eco*RV and *Ase*I) used to introduce the LoxP sites. Middle panel: *Dnmt3a* flox locus (Nguyen *et al.* 2007) including relative positions of the LoxP sites (red triangles) flanking exons 18 through 20 and the residual Frt site (white triangle). Bottom panel: *Dnmt3a*-null locus that depicts the deletion of exons 18–20. (B) Analysis of *Dnmt3a* deletion efficiency in naïve B cells by qPCR (*N* = 5 per group). (C) Quantification of the percent of pro B (ProB), pre B (PreB), and immature B (ImmB) cell populations in the bone marrow and (D) quantification of the percent of B cell, CD4^+^ T cell (CD4^+^CD8^−^), and CD8^+^ T cell (CD4^−^ CD8^+^) populations in the spleen for *Dnmt3a^+/+^* (gray bars) and *Dnmt3a^−/−^* (yellow bars) mice (*N* = 3 per group). Error bars indicate SEM.

A conditional knockout allele was used to target the *Dnmt3a* gene, where exons encoding the catalytic domain (exons 18–20) of *Dnmt3a* were flanked by loxP sites (Nguyen *et al.* 2007), in conjunction with the Cre-loxP recombination system (Figure 1A). *Dnmt3a^flox/flox^* mice were bred to mice containing the *Cd19-Cre* allele (*Cd19^Cre/+^*) (Rickert *et al.* 1997) to selectively inactivate the *Dnmt3a* gene in B lymphocytes. The *Cd19* promoter specifically directs *Cre* expression at the earliest stages and throughout B lymphocyte development and differentiation. As compared to *Dnmt3a^flox/flox^* × *Cd19^+/+^* (*Dnmt3a^+/+^*) littermate control mice, a 91% mean deletion efficiency was observed in splenic naïve B cells (B220^+^IgD^++^GL7^−^) of *Dnmt3a^flox/flox^* × *Cd19^Cre/+^* (*Dnmt3a^−/−^*) mice (Figure 1B), which is within the known range for splenic B cell deletion efficiency associated with *Cd19-Cre*–mediated mutagenesis (Rickert *et al.* 1997).

To understand the phenotypic consequences of B cell-specific *Dnmt3a* deletion, we first characterized B cell maturation in the bone marrow by flow cytometry. The acute depletion of *Dnmt3a* (*Dnmt3a^−/−^*) did not disrupt the total number or frequency of developing B cells (pro B cells, pre B cells, immature B cells) as compared to littermate control mice (*Dnmt3a^+/+^*, Figure 1C). In addition, the depletion of *Dnmt3a* did not affect the total number or frequency of lymphocytes (B cells, T cells) in the spleen (Figure 1D). Further, characterization of lymphocyte populations in the spleen showed no change in the ratio of B to T (CD4^+^ or CD8^+^) cells in *Dnmt3a*-deficient mice as compared to control mice. Thus, we conclude that B cell development is not grossly affected by deletion of *Dnmt3a*, and since B cells develop normally, we are able to study the effects of *Dnmt3a* on downstream DNA methylation patterns.

### Base-resolution methylomes of Dnmt3a knockout splenic naïve B cells

To assess the effect of *Dnmt3a* on genome-wide DNA methylation patterns, we generated base-resolution methylomes using WGBS for splenic naïve B cells (B220^+^IgD^++^GL7^−^) from adult *Dnmt3a^+/+^* and *Dnmt3a^−/−^* mice. To minimize bias caused by a biological outlier, multiple animals were used, and libraries from three independent animals per genotype were sequenced to a total depth of 20–30× per genotype, as is common practice in the field (Yang *et al.* 2016; Kawakatsu *et al.* 2017). To control for variations in genetic background and to exclude polymorphic CpG sites in our mixed genetic background model (B6;129), a genomic context validation strategy was implemented to restrict downstream analyses to mm9 CpG sites that occur in CpG context in all samples (see *Materials and Methods*). A total of 18,312,876 out of 21,722,957 (84.3%) CpG sites passed the genomic context validation criteria and were utilized for downstream analyses. For *Dnmt3a^+/+^* and *Dnmt3a^−/−^* cells, at least 91.2% of validated CpG sites have a read depth of at least 10 (Figure S1). A summary of sequence alignment and processing metrics is included in Table S1 of File S1.

### B cell-specific deletion of Dnmt3a does not globally alter DNA methylation distribution in splenic naïve B cells

**Figure 2:**
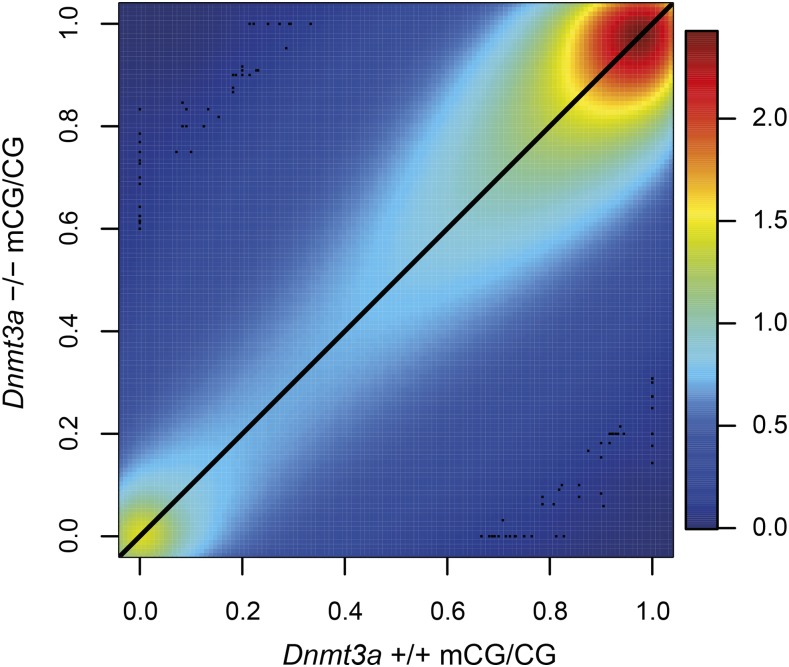
Comparison of single-site CpG methylation levels in splenic naïve B cells with and without *Dnmt3a*. Smoothed kernel density scatterplot of *Dnmt3a^+/+^* mCG/CG (*x*-axis) *vs. Dnmt3a^−/−^* mCG/CG (*y*-axis) for all validated CpG sites with read depth of at least 10 in both groups (*N* = 16,233,681). Points to the upper left of the diagonal reflect CpGs with greater methylation in *Dnmt3a^−/−^* and points to the lower right of the diagonal reflect CpGs with greater methylation in *Dnmt3a^+/+^*.

Using the base-resolution methylomes, we first asked if B cell-specific deletion of *Dnmt3a* effects DNA methylation levels at a global scale in splenic naïve B cells. The baseline distribution of CpG methylation in wild-type mouse naïve B cells was largely bimodal, and the vast majority of CpG sites were found to be methylated (Figure S2A), consistent with reported methylomes of sorted human naïve B cells (Kulis *et al.* 2015). We found global methylation distributions to be extremely consistent between *Dnmt3a^+/+^* and *Dnmt3a^−/−^* B cells (Figure S2, A and B). In both *Dnmt3a^+/+^* and *Dnmt3a^−/−^* B cells, 82% of validated CpG sites with read depth of at least 10 were largely methylated (mCG/CG > 0.75) whereas 7% were largely unmethylated (mCG/CG < 0.25). CpGs with intermediate methylation level (mCG/CG between 0.25 and 0.75) accounted for 11% of all validated sites assayed in each sample. Comparison of single-CpG methylation levels across the genome in naïve B cells with and without *Dnmt3a* confirmed that knockout of *Dnmt3a* does not induce a gross global shift of DNA methylation, as site-specific mCG/CG values exhibit a strong positive linear relationship between *Dnmt3a^+/+^* and *Dnmt3a^−/−^* B cells (Figure 2).

### B cell-specific deletion of Dnmt3a induces a limited number of site-specific DNA methylation alterations in splenic naïve B cells

**Figure 3:**
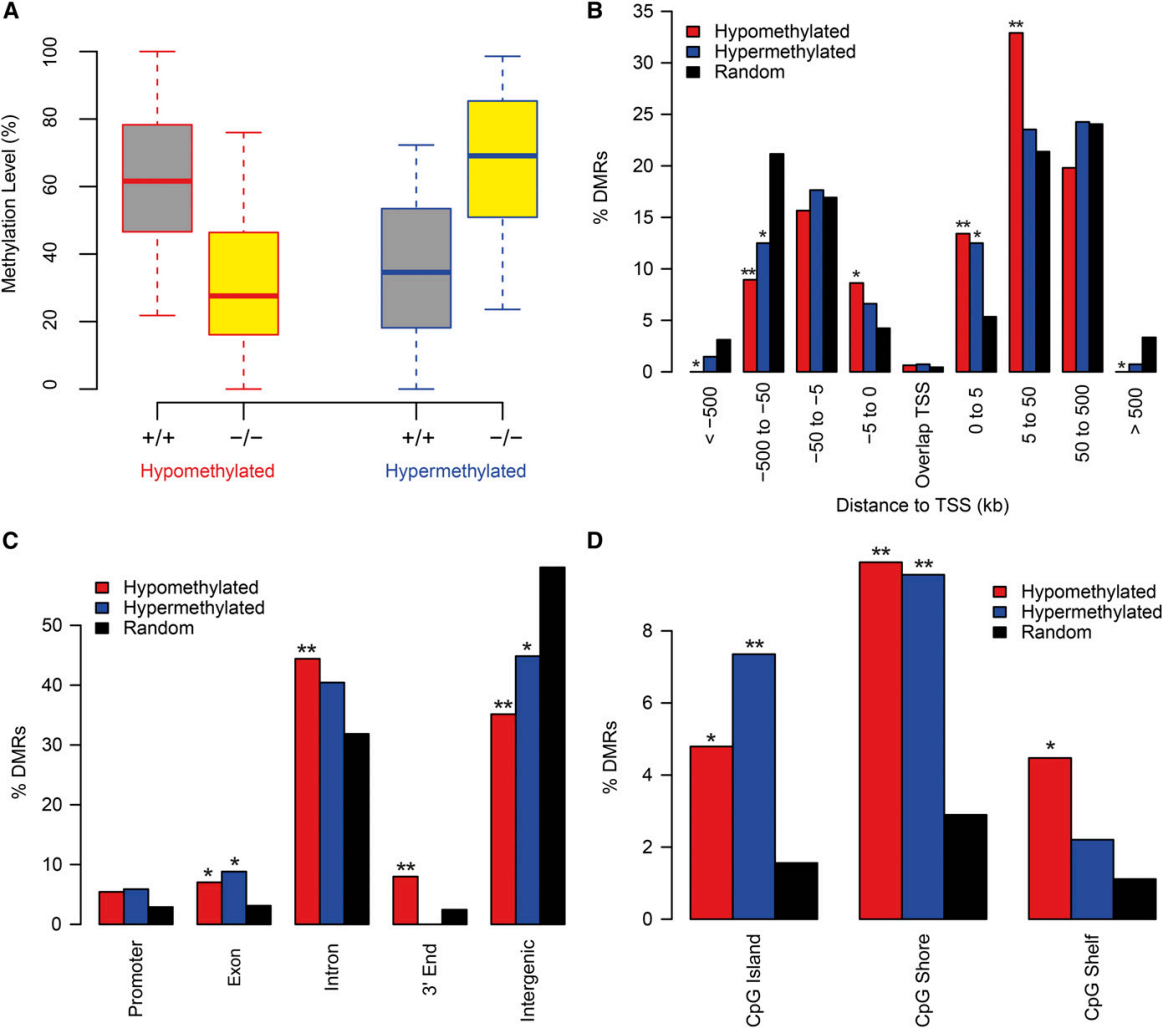
Summary characteristics of *Dnmt3a*-dependent DMRs. (A) Methylation levels at *Dnmt3a*-dependent DMRs by direction of methylation change. Box and whisker plots represent methylation levels for *Dnmt3a^+/+^* (gray fill) and *Dnmt3a^−/−^* (yellow fill) B cells at hypomethylated DMRs (red outline) and hyper-methylated DMRs (blue outline). (B) Percent of DMRs that fall in distance-dependent bins relative to the single nearest TSS. (C) Percent of DMRs that fall in gene model contexts for promoter, exon, intron, 3′ end, and intergenic regions. (D) Percent of DMRs that overlap with CpG-islands (UCSC genome browser), CpG-shores (±2 kb from islands), and CpG shelves (±2 kb from shores). Hypomethylated (red) and hypermethylated (blue) DMRs are compared with a length-matched random genomic null region set (black). Significant differences from the random set are noted; * *P* < 0.05, ** *P* < 0.001, two-sample Z-test for the difference in proportions.

We next asked if B cell-specific deletion of *Dnmt3a* induces focal, site-specific DNA methylation alterations in splenic naïve B cells. To define regions that are differentially methylated between *Dnmt3a^+/+^* and *Dnmt3a^−/−^* B cells, we utilized the differential methylation analysis algorithm, MOABS (Model-based Analysis of Bisulfite Sequencing data) (Sun *et al.* 2014). In total, 449 DMRs were identified, and both hypomethylated DMRs (Table S2 in File S1) and hypermethylated DMRs (Table S3 in File S1) were identified. However, regions hypomethylated in *Dnmt3a^−/−^* cells (*N* = 313) were over twofold more frequently observed than hypermethylated regions (*N* = 136), consistent with loss of methyltransferase activity. Loci that became hypo- or hypermethylated in *Dnmt3a^−/−^* cells were found to occur at regions spanning a broad range of methylation levels in *Dnmt3a^+/+^* cells, specifically 22–100% for hypomethylated loci and 0–76% for hypermethylated loci (Figure 3A). At the majority of DMRs, intermediate regional methylation levels are observed in both *Dnmt3a^+/+^* and *Dnmt3a^−/−^* cells. Together, the relatively small number of DMRs occurring in both directions (hypo- and hypermethylation) across a broad range of methylation levels are consistent with the lack of an observed systematic effect on global methylation distribution.

However, the hypo- and hypermethylated DMRs exhibit differential distributions relative to genomic context. The percentage of hyper-methylated DMRs that fall in distance-dependent bins relative to the nearest TSS closely resemble the percentage of length-matched random genomic null regions (Figure 3B). However, hypermethylated DMRs exhibited significant enrichment within 5 kb downstream of TSSs (Figure 3B). Alternatively, hypomethylated DMRs were highly enriched between 0 and 50 kb downstream of TSSs. Further, hypomethylated DMRs were notably depleted at TSS-distal regions and were completely absent in gene-poor regions, *i.e.*, >500 kb away from the nearest TSS (Figure 3B). Likewise, hypomethylated DMRs were enriched in gene body elements, including exons, introns, and 3′-ends while being highly depleted in intergenic regions (Figure 3C). Consistent with enrichment of both hypo- and hypermethylated DMRs within 5 kb of TSSs, both were highly enriched near CpG islands, particularly at CpG shores (Figure 3D). Together, these results support a model in which B cell-specific methylation by DNMT3A is targeted to gene-centric regions.

### Dnmt3a^−/−^-associated hypomethylation targets genes involved in B cell-related phenotypes and disease

**Figure 4:**
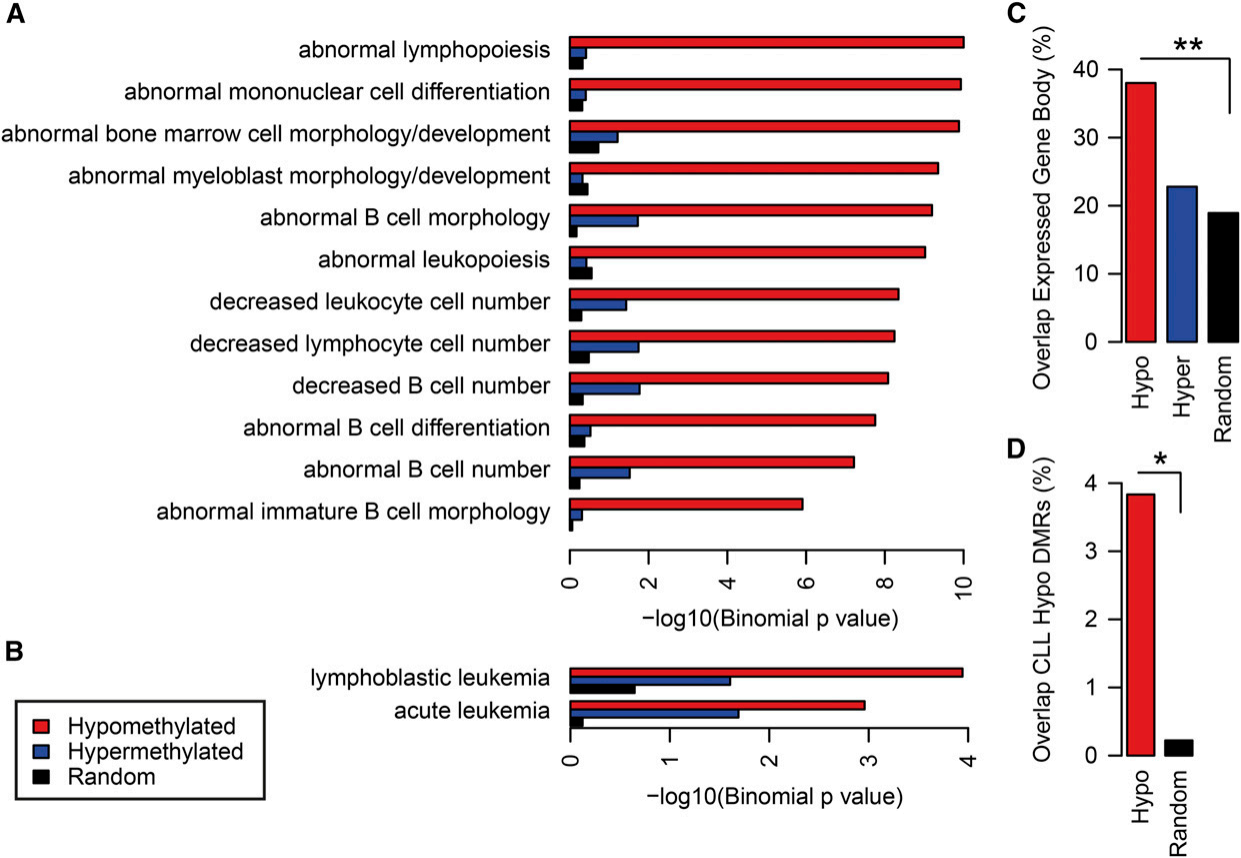
Hypomethylated *Dnmt3a*-dependent DMRs are located in and around genes with functional roles in B cell development and disease. Statistical enrichments for association between putative target genes of *Dnmt3a*-dependent DMRs and annotations of the (A) Mouse Genome Informatics (MGI) Phenotype Ontology and (B) the Disease Ontology. The *x*-axis corresponds to raw *P* values (binomial test, in logarithmic scale). (C) Percent overlap of hypomethylated (red) and hypermethylated (blue) DMRs with gene bodies of transcripts expressed in B cells, as compared with length-matched random genomic null regions (black). (D) Percent overlap of hypomethylated DMRs (red) with hypomethylated promoter and gene body DMRs in Dnmt3a^Δ/Δ^ CLL relative to B-1a cells (Haney *et al.* 2016a), as compared with length-matched random genomic null regions (black). Significant differences from the random set are noted; * *P* = 1.5 × 10^−4^, ** *P* = 4.8 × 10^−9^, two-sample Z-test for the difference in proportions.

To gain insight on the functional significance of genes associated with *Dnmt3a*-dependent hypo- and hypermethylation events, we performed gene ontology analyses using GREAT (McLean *et al.* 2010), which enables prediction of function for *cis*-regulatory regions. Interestingly, statistical enrichments for association between putative target genes of *Dnmt3a*-dependent hypomethylated DMRs and annotations of the MGI Phenotype Ontology (Blake *et al.* 2009) indicated significant links to B cell-related mouse phenotypes such as abnormal B cell morphology, cell number, and differentiation (Figure 4A). Conversely, significant enrichments of B cell-related mouse phenotypes were not observed for genes associated with hypermethylated DMRs or random null control regions. Furthermore, upon examination of associations with terms of the Disease Ontology, a standardized ontology for human disease, putative target genes of *Dnmt3a*-dependent hypomethylated DMRs were found to be enriched for genes involved in lymphoblastic leukemia, a term including the B cell malignancy, CLL (Figure 4B). Again, functional enrichment of B cell-related phenotypes and disease were solely driven by hypomethylated DMRs, rather than hypermethylated DMRs.

Based on the functional enrichment of *Dnmt3a*-dependent DMRs in B cell-related phenotypes and on previous work supporting the role of DNMT3A in catalyzing DNA methylation in gene bodies of transcriptionally permissive genes (Wu *et al.* 2010), we hypothesized that in B cell development, DNMT3A is likely catalyzing DNA methylation in gene bodies of transcripts expressed in B cells. To test this hypothesis, we first utilized existing RNA-seq data for follicular B cells (small B220^+^CD21^+^CD23^+^) (Shi *et al.* 2015), which represent the majority of naive splenic B cells, to define the set of genes expressed in B cells. Next, we asked whether *Dnmt3a*-dependent DMRs overlap gene bodies, defined as the region from the TSS to the TES, of B cell-expressed genes. Indeed, nearly 40% of hypomethylated DMRs were found to overlap gene bodies of transcripts expressed in B cells. Hypomethylated DMRs were found to overlap B cell-expressed gene bodies approximately twofold more frequently than hypermethylated regions or a length-matched random genomic null region set (Figure 4C).

Next, we asked to what extent do the *Dnmt3a*-dependent DMRs observed here overlap with DNA methylation alterations observed in a mouse model of *Dnmt3a*-null CLL. To do this, we assessed DMRs recently reported by Haney *et al.*, contrasting splenic B-1a cells from a *Dnmt3a*^+/+^ control mouse and a EμSRα-tTA;Teto-Cre;Dnmt3a^fl/fl^;Rosa26LOXP^EGFP/EGFP^ (*Dnmt3a*^Δ/Δ^) mouse with CLL (Haney *et al.* 2016a). Hypo- and hypermethylated *Dnmt3a*^Δ/Δ^ CLL DMRs located in promoter and gene body regions were compared with *Dnmt3a^−/−^* naïve B cell hypo- and hypermethylated DMRs, respectively. A total of ∼4% of hypomethylated *Dnmt3a^−/−^* naïve B cell DMRs directly overlapped with hypomethylated *Dnmt3a*^Δ/Δ^ CLL DMRs, which was significantly greater than the overlap observed with a random genomic null region set (Figure 4D). Conversely, no overlap was observed between the hypermethylated DMR sets. Together, these data indicate that the limited number of *Dnmt3a^−/−^*-associated hypomethylation events target genes involved in B cell-related phenotypes, primarily in gene bodies of transcripts expressed in B cells, a small fraction of which are associated with B cell malignancies.

## Discussion

Here, we report high-quality base-resolution CpG methylation profiles of *Dnmt3a^+/+^* and *Dnmt3a^−/−^* mouse naïve splenic B cells. Globally, the observed bimodal CpG methylation distribution in naïve B cells is largely consistent with features of the mouse ES cell methylome (Stadler *et al.* 2011) and with previously reported genome-wide profiles in human naïve B cells (Kulis *et al.* 2015). Similar to human uncommitted hematopoietic progenitor cells, pre-BII cells, and naïve B cells (Kulis *et al.* 2015), examination of global DNA methylation distributions revealed that the vast majority of CpG sites (82%) in mouse naïve splenic B cells are methylated, while only a small proportion (7%) are unmethylated. In contrast, DNA methylation is known to be remodeled in GC B cells, memory B cells, and plasma cells during a B cell immune response, and is characterized by hypomethylation and increased methylome diversity in conjunction with dynamic regulation of three methyltransferases: *Dnmt1*, *Dnmt3b*, and *Dnmt3a* (Barwick *et al.* 2016; Dominguez *et al.* 2015; Kulis *et al.* 2015; Lai *et al.* 2013; Shaknovich *et al.* 2011).

Examining the effects of *Dnmt3a* on DNA methylation patterns in B cell development, we generally observed modest changes in naïve B cells lacking *Dnmt3a*. Methylation changes downstream of B cell-specific *Dnmt3a* knockout occur at a focal scale rather than by affecting DNA methylation at a global scale. The observed DMRs represent a small fraction of the genome (<1%), which is consistent with our observation that B cells lacking *Dnmt3a* develop normally. However, while B cell-specific deletion of *Dnmt3a* affected only a small fraction of the genome, the regions which are differentially methylated reflect a connection to B cell biology.

Based on the data presented here, DNA methylation changes associated with B cell-specific *Dnmt3a* knockout most frequently correspond to hypomethylation, which is consistent with loss of DNMT activity. Further, the higher proportion of hypomethylation in naïve B cells is consistent with observations of hypomethylation in *DNMT3A*-mutant and *Dnmt3a*-null leukemias (Haney *et al.* 2016a,b; Russler-Germain *et al.* 2014; Yang *et al.* 2016). Contrary to observations of promoter hypomethylation in *Dnmt3a^+/−^* and *Dnmt3a^−/−^* CLL (Haney *et al.* 2016b), promoters were minimally affected in *Dnmt3a^−/−^* naïve B cells. This difference in outcomes may be influenced by DMRs resulting from the pathogenesis of CLL itself rather than loss of *Dnmt3a*.

The hypomethylation events observed in *Dnmt3a*-null B cells were enriched in gene bodies, particularly in bodies of genes that are poised for transcription in B cells. Correspondingly, gene-poor regions (gene deserts) are devoid of hypomethylated DMRs in *Dnmt3a^−/−^* cells. Together, this evidence is consistent with existing models that one function of DNMT3A is to methylate gene bodies (Wu *et al.* 2010). Hypermethylation events observed in this work were enriched in promoter proximal regions within gene bodies (Figure 4B). While the mechanistic basis of these events is currently unclear, it is conceivable that these loci are preferential targets for methylation by other DNMTs (including DNMT3B) that are revealed upon loss of DNMT3A activity.

Further, the results presented here allow us to speculate on comparisons of *Dnmt3a* loss-of-function in B cell-derived cells and early hematopoietic cell types. It is clear that DNMT3A is crucial to embryonic stem (ES) cell and hematopoietic stem cell (HSC) differentiation and regulates the function of stem cells. *DNMT3A* mutations occur in human HSCs, in which they act as a preleukemic lesion (Yang *et al.* 2015). In the mouse, loss of *Dnmt3a* in ES cells progressively impairs HSC differentiation over serial transplantation, causes HSC expansion (Challen *et al.* 2011), and predisposes HSCs to malignant transformation (Mayle *et al.* 2015). Further, conditional inactivation of *Dnmt3a* in HSCs and progenitors results in the development of CLL (Peters *et al.* 2014). The functional and epigenomic data here are consistent with the hypothesis that the cells of origin for these tumors are early hematopoietic cell types rather than B cell-derived descendants.

*Dnmt3a*, in conjunction with *Dnmt1* and *Dnmt3b*, is known to be dynamically regulated throughout normal B cell development and during a B cell immune response, and its inactivation in hematopoietic stem cells has been shown to drive B cell-related malignancies. Here, we tested whether B cell-specific loss of *Dnmt3a* leads to DNA methylation defects in B cells that might impair function. While we identified a subset of CpGs that require *Dnmt3a* to maintain DNA methylation patterns in naïve B cells, we do not observe DNA methylation changes that would indicate large functional changes and predict that phenotypic consequences would be minimal. The results from this study suggest that factors other than *Dnmt3a* are the major drivers for methylome maintenance in B cell development.

## Supplementary Material

Supplemental material is available online at www.g3journal.org/lookup/suppl/doi:10.1534/g3.117.300446/-/DC1.

## References

[bib1] Barau J. Teissandier A. Zamudio N. Roy S. Nalesso V., 2016 The DNA methyltransferase DNMT3C protects male germ cells from transposon activity. Science 354: 909–912. 27856912 10.1126/science.aah5143

[bib2] Barwick B. G. Scharer C. D. Bally A. P. Boss J. M., 2016 Plasma cell differentiation is coupled to division-dependent DNA hypomethylation and gene regulation. Nat. Immunol. 17: 1216–1225. 27500631 10.1038/ni.3519 PMC5157049

[bib3] Bhasin J. M. Ting A. H., 2016 Goldmine integrates information placing genomic ranges into meaningful biological contexts. Nucleic Acids Res. 44: 5550–5556. 27257071 10.1093/nar/gkw477 PMC4937336

[bib4] Bibikova M. Barnes B. Tsan C. Ho V. Klotzle B., 2011 High density DNA methylation array with single CpG site resolution. Genomics 98: 288–295. 21839163 10.1016/j.ygeno.2011.07.007

[bib5] Blake J. A. Bult C. J. Eppig J. T. Kadin J. A. Richardson J. E., 2009 The mouse genome database genotypes:phenotypes. Nucleic Acids Res. 37: D712–D719. 18981050 10.1093/nar/gkn886 PMC2686566

[bib6] Blanco-Betancourt C. E. Moncla A. Milili M. Jiang Y. L. Viegas-Pequignot E. M., 2004 Defective B-cell-negative selection and terminal differentiation in the ICF syndrome. Blood 103: 2683–2690. 14645008 10.1182/blood-2003-08-2632

[bib7] Brunetti L. Gundry M. C. Goodell M. A., 2017 DNMT3A in leukemia. Cold Spring Harb. Perspect. Med. 7: a030320. 28003281 10.1101/cshperspect.a030320 PMC5287058

[bib8] Cabezas-Wallscheid N. Klimmeck D. Hansson J. Lipka D. B. Reyes A., 2014 Identification of regulatory networks in HSCs and their immediate progeny via integrated proteome, transcriptome, and DNA methylome analysis. Cell Stem Cell 15: 507–522. 25158935 10.1016/j.stem.2014.07.005

[bib9] Challen G. A. Sun D. Jeong M. Luo M. Jelinek J., 2011 Dnmt3a is essential for hematopoietic stem cell differentiation. Nat. Genet. 44: 23–31. 22138693 10.1038/ng.1009 PMC3637952

[bib10] Doi A. Park I. H. Wen B. Murakami P. Aryee M. J., 2009 Differential methylation of tissue- and cancer-specific CpG island shores distinguishes human induced pluripotent stem cells, embryonic stem cells and fibroblasts. Nat. Genet. 41: 1350–1353. 19881528 10.1038/ng.471 PMC2958040

[bib11] Dominguez P. M. Teater M. Chambwe N. Kormaksson M. Redmond D., 2015 DNA methylation dynamics of germinal center B cells are mediated by AID. Cell Rep. 12: 2086–2098. 26365193 10.1016/j.celrep.2015.08.036 PMC4591215

[bib12] Edgar R. Domrachev M. Lash A. E., 2002 Gene expression omnibus: NCBI gene expression and hybridization array data repository. Nucleic Acids Res. 30: 207–210. 11752295 10.1093/nar/30.1.207 PMC99122

[bib13] Haney S. L. Upchurch G. M. Opavska J. Klinkebiel D. Appiah A. K., 2016 a Loss of Dnmt3a induces CLL and PTCL with distinct methylomes and transcriptomes in mice. Sci. Rep. 6: 34222. 27677595 10.1038/srep34222 PMC5039761

[bib14] Haney S. L. Upchurch G. M. Opavska J. Klinkebiel D. Hlady R. A., 2016 b Promoter hypomethylation and expression is conserved in mouse chronic lymphocytic leukemia induced by decreased or inactivated Dnmt3a. Cell Rep. 15: 1190–1201. 27134162 10.1016/j.celrep.2016.04.004 PMC4864108

[bib15] Holliday R. Pugh J. E., 1975 DNA modification mechanisms and gene activity during development. Science 187: 226–232. 1111098

[bib16] Irizarry R. A. Ladd-Acosta C. Wen B. Wu Z. Montano C., 2009 The human colon cancer methylome shows similar hypo- and hypermethylation at conserved tissue-specific CpG island shores. Nat. Genet. 41: 178–186. 19151715 10.1038/ng.298 PMC2729128

[bib17] Jones P. A. Liang G., 2009 Rethinking how DNA methylation patterns are maintained. Nat. Rev. Genet. 10: 805–811. 19789556 10.1038/nrg2651 PMC2848124

[bib18] Karolchik D. Hinrichs A. S. Furey T. S. Roskin K. M. Sugnet C. W., 2004 The UCSC table browser data retrieval tool. Nucleic Acids Res. 32: D493–D496. 14681465 10.1093/nar/gkh103 PMC308837

[bib19] Kawakatsu T. Nery J. R. Castanon R. Ecker J. R., 2017 Dynamic DNA methylation reconfiguration during seed development and germination. Genome Biol. 18: 171. 28911331 10.1186/s13059-017-1251-x PMC5599895

[bib20] Krueger F. Andrews S. R., 2011 Bismark: a flexible aligner and methylation caller for Bisulfite-Seq applications. Bioinformatics 27: 1571–1572. 21493656 10.1093/bioinformatics/btr167 PMC3102221

[bib21] Kulis M. Merkel A. Heath S. Queiros A. C. Schuyler R. P., 2015 Whole-genome fingerprint of the DNA methylome during human B cell differentiation. Nat. Genet. 47: 746–756. 26053498 10.1038/ng.3291 PMC5444519

[bib22] Lai A. Y. Mav D. Shah R. Grimm S. A. Phadke D., 2013 DNA methylation profiling in human B cells reveals immune regulatory elements and epigenetic plasticity at Alu elements during B-cell activation. Genome Res. 23: 2030–2041. 24013550 10.1101/gr.155473.113 PMC3847773

[bib23] Langmead B. Trapnell C. Pop M. Salzberg S. L., 2009 Ultrafast and memory-efficient alignment of short DNA sequences to the human genome. Genome Biol. 10: R25. 19261174 10.1186/gb-2009-10-3-r25 PMC2690996

[bib24] Lara-Astiaso D. Weiner A. Lorenzo-Vivas E. Zaretsky I. Jaitin D. A., 2014 Immunogenetics. Chromatin state dynamics during blood formation. Science 345: 943–949. 25103404 10.1126/science.1256271 PMC4412442

[bib25] Leonhardt H. Page A. W. Weier H. U. Bestor T. H., 1992 A targeting sequence directs DNA methyltransferase to sites of DNA replication in mammalian nuclei. Cell 71: 865–873. 1423634 10.1016/0092-8674(92)90561-p

[bib26] Li E. Bestor T. H. Jaenisch R., 1992 Targeted mutation of the DNA methyltransferase gene results in embryonic lethality. Cell 69: 915–926. 1606615 10.1016/0092-8674(92)90611-f

[bib27] Mayle A. Yang L. Rodriguez B. Zhou T. Chang E., 2015 Dnmt3a loss predisposes murine hematopoietic stem cells to malignant transformation. Blood 125: 629–638. 25416277 10.1182/blood-2014-08-594648 PMC4304108

[bib28] McLean C. Y. Bristor D. Hiller M. Clarke S. L. Schaar B. T., 2010 GREAT improves functional interpretation of cis-regulatory regions. Nat. Biotechnol. 28: 495–501. 20436461 10.1038/nbt.1630 PMC4840234

[bib29] Mudunuri U. Che A. Yi M. Stephens R. M., 2009 bioDBnet: the biological database network. Bioinformatics 25: 555–556. 19129209 10.1093/bioinformatics/btn654 PMC2642638

[bib30] National Research Council (US), 2011 *Guide for the Care and Use of Laboratory Animals*. National Academies Press, Washington, DC.

[bib31] Nguyen S. Meletis K. Fu D. Jhaveri S. Jaenisch R., 2007 Ablation of de novo DNA methyltransferase Dnmt3a in the nervous system leads to neuromuscular defects and shortened lifespan. Dev. Dyn. 236: 1663–1676. 17477386 10.1002/dvdy.21176

[bib32] Oakes C. C. Seifert M. Assenov Y. Gu L. Przekopowitz M., 2016 DNA methylation dynamics during B cell maturation underlie a continuum of disease phenotypes in chronic lymphocytic leukemia. Nat. Genet. 48: 253–264. 26780610 10.1038/ng.3488 PMC4963005

[bib33] Okano M. Bell D. W. Haber D. A. Li E., 1999 DNA methyltransferases Dnmt3a and Dnmt3b are essential for de novo methylation and mammalian development. Cell 99: 247–257. 10555141 10.1016/s0092-8674(00)81656-6

[bib34] O’Leary N. A. Wright M. W. Brister J. R. Ciufo S. Haddad D., 2016 Reference sequence (RefSeq) database at NCBI: current status, taxonomic expansion, and functional annotation. Nucleic Acids Res. 44: D733–D745. 26553804 10.1093/nar/gkv1189 PMC4702849

[bib35] Osborne J. D. Flatow J. Holko M. Lin S. M. Kibbe W. A., 2009 Annotating the human genome with disease ontology. BMC Genomics 10(Suppl. 1): S6. 10.1186/1471-2164-10-S1-S6 PMC2709267 19594883

[bib36] Peters S. L. Hlady R. A. Opavska J. Klinkebiel D. Pirruccello S. J., 2014 Tumor suppressor functions of Dnmt3a and Dnmt3b in the prevention of malignant mouse lymphopoiesis. Leukemia 28: 1138–1142. 24292811 10.1038/leu.2013.364

[bib37] Quinlan A. R. Hall I. M., 2010 BEDTools: a flexible suite of utilities for comparing genomic features. Bioinformatics 26: 841–842. 20110278 10.1093/bioinformatics/btq033 PMC2832824

[bib38] R Core Team, 2016 *R: A Language and Environment for Statistical Computing*. R Foundation for Statistical Computing, Vienna.

[bib39] Rickert R. C. Roes J. Rajewsky K., 1997 B lymphocyte-specific, Cre-mediated mutagenesis in mice. Nucleic Acids Res. 25: 1317–1318. 9092650 10.1093/nar/25.6.1317 PMC146582

[bib40] Riggs A. D., 1975 X inactivation, differentiation, and DNA methylation. Cytogenet. Cell Genet. 14: 9–25. 1093816 10.1159/000130315

[bib41] Russler-Germain D. A. Spencer D. H. Young M. A. Lamprecht T. L. Miller C. A., 2014 The R882H DNMT3A mutation associated with AML dominantly inhibits wild-type DNMT3A by blocking its ability to form active tetramers. Cancer Cell 25: 442–454. 24656771 10.1016/j.ccr.2014.02.010 PMC4018976

[bib42] Shaknovich R. Cerchietti L. Tsikitas L. Kormaksson M. De S., 2011 DNA methyltransferase 1 and DNA methylation patterning contribute to germinal center B-cell differentiation. Blood 118: 3559–3569. 21828137 10.1182/blood-2011-06-357996 PMC3186332

[bib43] Shi W. Liao Y. Willis S. N. Taubenheim N. Inouye M., 2015 Transcriptional profiling of mouse B cell terminal differentiation defines a signature for antibody-secreting plasma cells. Nat. Immunol. 16: 663–673. 25894659 10.1038/ni.3154

[bib44] Stadler M. B. Murr R. Burger L. Ivanek R. Lienert F., 2011 DNA-binding factors shape the mouse methylome at distal regulatory regions. Nature 480: 490–495. 22170606 10.1038/nature10716

[bib45] Sun D. Xi Y. Rodriguez B. Park H. J. Tong P., 2014 MOABS: model based analysis of bisulfite sequencing data. Genome Biol. 15: R38. 24565500 10.1186/gb-2014-15-2-r38 PMC4054608

[bib46] Wu H. Coskun V. Tao J. Xie W. Ge W., 2010 Dnmt3a-dependent nonpromoter DNA methylation facilitates transcription of neurogenic genes. Science 329: 444–448. 20651149 10.1126/science.1190485 PMC3539760

[bib47] Yang L. Rau R. Goodell M. A., 2015 DNMT3A in haematological malignancies. Nat. Rev. Cancer 15: 152–165. 25693834 10.1038/nrc3895 PMC5814392

[bib48] Yang L. Rodriguez B. Mayle A. Park H. J. Lin X., 2016 DNMT3A loss drives enhancer hypomethylation in FLT3-ITD-associated leukemias. Cancer Cell 29: 922–934 (erratum: Cancer Cell 30: 363–365). 27300438 10.1016/j.ccell.2016.05.003 PMC4908977

