# Supplementary material for: Base-Resolution Analysis of DNA Methylation Patterns Downstream of Dnmt3a in Mouse Naïve B Cells
Source: G3 (Bethesda). 2018 Jan 11;8(3):805–13. doi: 10.1534/g3.117.300446 (PMC5844302; doi:10.1534/g3.117.300446)
Supplement: Supplementary file 1 [file 805FigureS1.pdf]

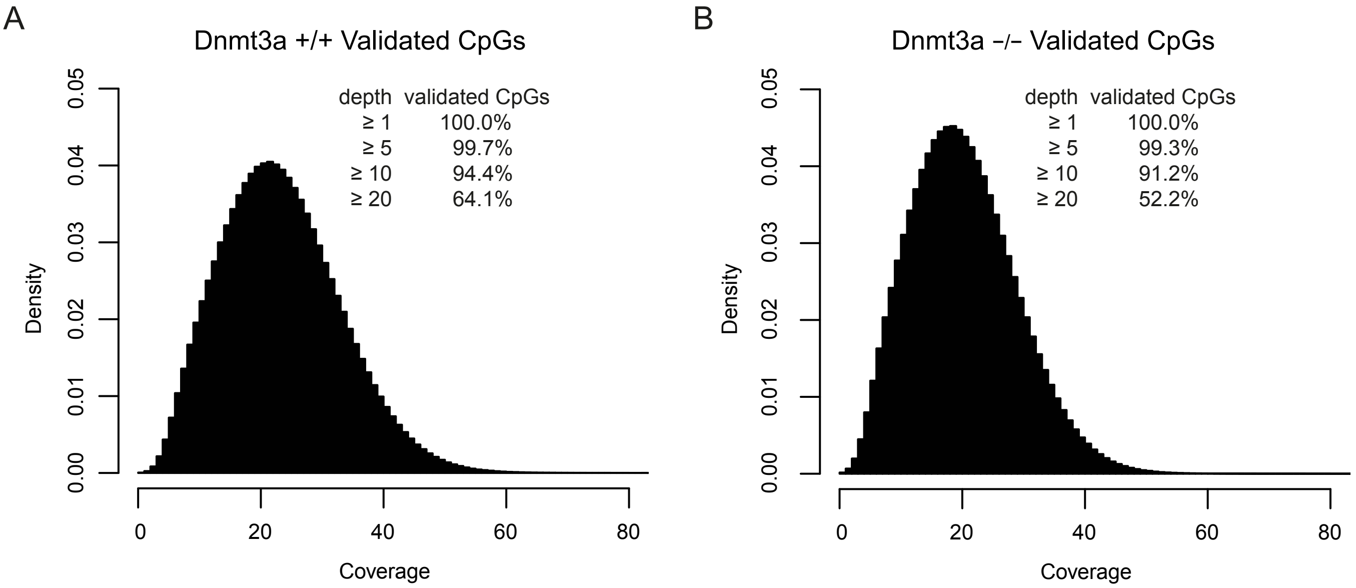


**Figure S1** – Sequencing coverage for validated CpG sites. Read depth distribution of validated CpG sites for (A) *Dnmt3a*+/+ and (B) *Dnmt3a*−/− naïve B cells. Read depth is calculated based on merged strand data after all alignment, trimming, and filtering steps. Inset table lists percent of validated CpG sites exceeding given read depth thresholds for each sample.
