# Supplementary material for: Base-Resolution Analysis of DNA Methylation Patterns Downstream of Dnmt3a in Mouse Naïve B Cells
Source: G3 (Bethesda). 2018 Jan 11;8(3):805–13. doi: 10.1534/g3.117.300446 (PMC5844302; doi:10.1534/g3.117.300446)
Supplement: Supplementary file 2 [file 805FigureS2.pdf]

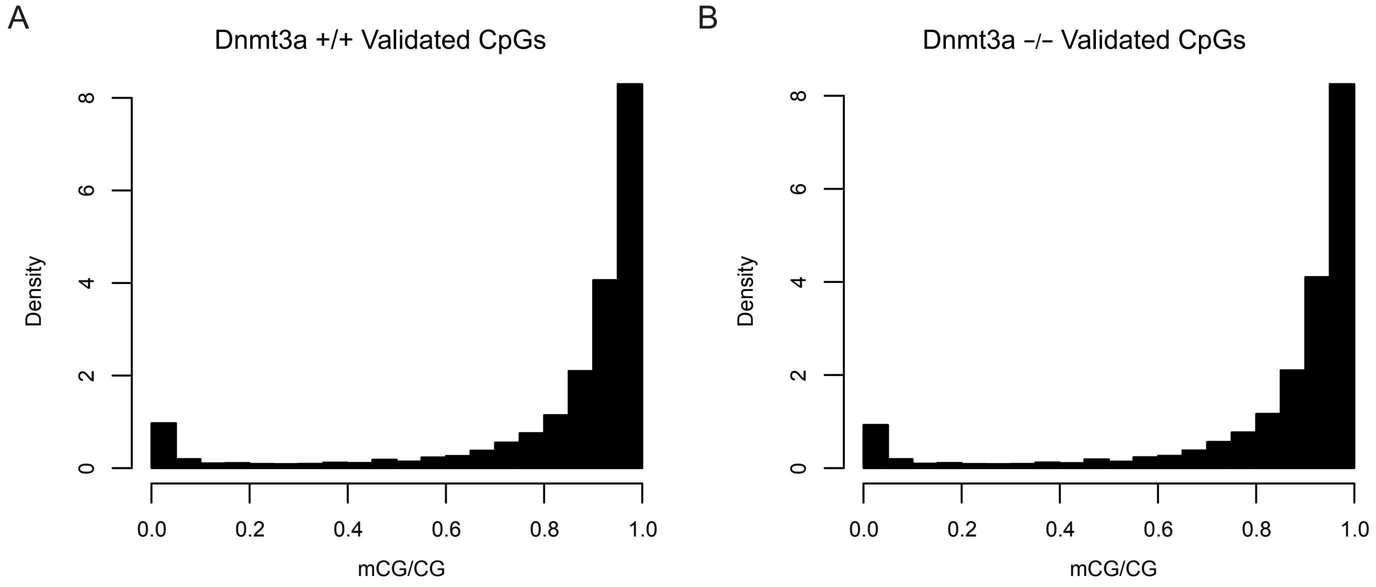

Figure S2 – Global density distribution of single-site CpG methylation levels in splenic naïve B cells with and without *Dnmt3a*. Density distribution (bin size, 0.05) of single-site mCG/CG levels for all validated CpG sites with read depth of at least 10 for (A) *Dnmt3a*+/+ (N = 17,295,282) and (B) *Dnmt3a*−/− (N = 16,696,664) samples.
